# Genome Sequences of Five Novel Neisseria gonorrhoeae Sequence Types Isolated in KwaZulu-Natal, South Africa

**DOI:** 10.1128/MRA.01424-20

**Published:** 2021-03-04

**Authors:** Nireshni Mitchev, Mushal Allam, Stanford Kwenda, Florah Mnyameni, Arshad Ismail, Abraham J. Niehaus, Veron Ramsuran, Nigel Garrett, Ravesh Singh, Koleka P. Mlisana

**Affiliations:** a School of Laboratory Medicine and Medical Sciences, University of KwaZulu-Natal, Durban, South Africa; b Sequencing Core Facility, National Institute for Communicable Diseases, National Health Laboratory Service, Johannesburg, South Africa; c Centre for the AIDS Programme of Research in South Africa, Durban, South Africa; d School of Nursing and Public Health, Discipline of Public Health Medicine, UKZN, Durban, South Africa; e National Health Laboratory Service, Durban, South Africa; University of Maryland School of Medicine

## Abstract

Africa has the highest incidence of Neisseria gonorrhoeae infections globally, but data on these isolates is scarce. Here, we report six N. gonorrhoeae genome sequences with five novel sequence types isolated from patients with uncomplicated genitourinary gonorrhea in South Africa.

## ANNOUNCEMENT

Neisseria gonorrhoeae, a Gram-negative diplococcus, is an obligate human pathogen which infects the mucosal epithelium of the genitourinary tract and anorectal and pharyngeal mucosal surfaces (1, 2). Untreated gonorrhea can lead to numerous adverse events, including acute urethritis, cervicitis, pelvic inflammatory disease, infertility, abortion, ectopic pregnancy, and maternal death, and in neonates, gonorrheal infection may lead to blindness (3–7). We are in the era of encountering untreatable N. gonorrhoeae, and thus, continued surveillance at the regional, national, and international levels is required to monitor and inform treatment. In this announcement, we characterize the whole-genome sequences of six N. gonorrhoeae isolates with five novel sequence types (STs) isolated from urethral or vaginal swabs of clinic patients in KwaZulu-Natal, South Africa.

N. gonorrhoeae clinical isolates from the University of KwaZulu-Natal repository collection were revived to determine the sequence types circulating in our region. Isolates were grown on nonselective Thayer Martin medium (supplemented with 1% Vitox) for 18 to 24 h in a 37°C, 5% CO_2_ incubator and confirmed using bright field microscopy, a Bactident oxidase rapid test (Merck, Germany), and a Phadebact monoclonal GC test (Pharmacia, Sweden). DNA was extracted using a PureLink microbiome DNA purification kit (Thermo Fisher, USA). Paired-end libraries were prepared using the Nextera DNA prep kit, followed by sequencing (2 × 75 bp) on the NextSeq platform (Illumina, Inc., USA). For bacterial whole-genome sequence analysis and typing, the JEKESA pipeline (https://github.com/stanikae/jekesa) was used. Briefly, Trim Galore v0.6.2 (https://github.com/FelixKrueger/TrimGalore) was used to filter the sequence reads (Q, ≥ 20; length, ≥ 50), *de novo* assembly was performed using SPAdes v3.13.2 (https://github.com/ablab/spades), the assemblies were polished and/or optimized using Shovill v1.1.0 (https://github.com/tseemann/shovill), and sequence typing was done using the multilocus sequence typing (MLST) tool v2.16.4 (https://github.com/tseemann/mlst). Assembly metrics, including the GC content and number of contigs, were calculated using QUAST v5.0.2 (http://quast.sourceforge.net/quast). All resultant contiguous sequences were annotated using the NCBI Prokaryotic Genome Annotation Pipeline v4.13 (8). Furthermore, the assembled genome sequences were uploaded to the Neisseria PubMLST database (https://pubmlst.org/organisms/neisseria-spp) to assign the novel sequence type.

The sequencing of the six isolates yielded an average of 3,750,403 raw reads. The high-quality reads (average, 3,570,038 reads) were assembled to contigs with an average of 141 contigs longer than 200 bp. The draft genome sequences for the six isolates were then submitted to Neisseria PubMLST, which assigned 5 novel STs (ST15652, ST15653, ST15654, ST15655, and ST15657). The key genomic features for the isolates are summarized in Table 1. Whole-genome sequence data from the WHO Africa region is scarce, and these South African N. gonorrhoeae genome sequences provide comprehensive information for surveillance and molecular epidemiology studies.

**TABLE 1:** Isolate information and key genomic features of the 6 N. gonorrhoeae isolates from KwaZulu-Natal, South Africa

Isolate name	SRA accession no.	BioSample accession no.	GenBank accession no.	PubMLST accession no.	MLST	No. of contigs	*N*_50_ (bp)	Genome length (bp)	Total no. of genes
ST11	SRR13300795	SAMN16967564	JADWTF000000000	96169	15652	139	48,332	2,103,486	2,187
ST25	SRR13300783	SAMN16967566	JADWVH000000000	96170	15653	146	41,065	2,111,095	2,207
SAR411	SRR13305402	SAMN16967583	JADWTR000000000	96171	15654	133	46,380	2,166,389	2,259
ST128	SRR13300787	SAMN16967585	JADWVL000000000	96172	15655	143	43,633	2,122,934	2,226
SAR54	SRR13305441	SAMN16967587	JADWTN000000000	96173	15655	142	41,076	2,164,406	2,279
SAR306	SRR13305407	SAMN16967593	JADWTW000000000	96174	15657	140	40,327	2,119,856	2,213

This study was approved by the University of KwaZulu-Natal Biomedical Research Ethics Committee (approval number BREC/00000097/2019).

### Data availability.

This whole-genome sequence project has been registered in DDBJ/ENA/GenBank with the BioProject accession number PRJNA681740. The GenBank, BioSample, SRA, and PubMLST accession numbers are provided in Table 1.
